# Facteurs associés à la pratique de l´allaitement maternel exclusif chez les mères d´enfants âgés de 6 à 12 mois dans la commune de Kaolack (Sénégal)

**DOI:** 10.11604/pamj.2023.45.55.39636

**Published:** 2023-05-24

**Authors:** Boubacar Gueye, Oumar Bassoum, Dieynaba Bassoum, Ndéye Marième Diagne, Martial Coly Bop, Alioune Badara Tall, Abdoul Aziz Ndiaye, Cheikh Tacko Diop, Papa Gallo Sow, Ousseynou Ka, Ibrahima Seck

**Affiliations:** 1 Université Alouine Diop de Bambeye, Unité de Formation et de Recherche, Santé et Développement Durable, Dakar, Sénégal,; 2 Université Cheikh Anta Diop de Dakar, Faculté de Médecine de Pharmacie et d´Odontostomatologie, Dakar, Sénégal,; 3 Institut Santé et Développement, Dakar, Sénégal,; 4 Hôpital Principal de Dakar, Dakar, Sénégal

**Keywords:** Allaitement maternel exclusif, facteurs associés, épidémiologie, Kaolack, Exclusive breastfeeding, associated factors, epidemiology, Kaolack

## Abstract

**Introduction:**

l´allaitement maternel immédiat et exclusif est l´une des interventions majeures recommandées par l´Organisation mondiale de la Santé (OMS) permettant de réduire la malnutrition et la mortalité néonatale. L´objectif de cette étude est de déterminer le taux de prévalence de la pratique de l´allaitement maternel exclusif (AME) et d´identifier ses facteurs associés chez les mères d´enfants âgés de 6 à 12 mois à Kaolack.

**Méthodes:**

c´est une étude transversale descriptive et analytique. La période d´étude s´étalait du 08 juillet 2020 au 08 février 2021. L´analyse multivariée a été faite grâce au modèle de régression logistique. La population cible était représentée par les mères d´enfants âgés de 6 à 12 mois résidant dans la ville de Kaolack rencontrées lors des séances de vaccination.

**Résultats:**

au total 400 femmes ont été enquêtées. Parmi elles 51,8% ont pratiqué l´AME selon les recommandations de l´OMS. L´âge moyen de ces mères est de 27,42 ans; 56,3% sont des femmes au foyer et 82,7% ont fait au moins 4 consultations prénatales (CPN). La régression logistique a mis en évidence une association entre l´AME et les facteurs suivants: l´âge < 25 ans (OR=2,03 IC 95% [1,23-3,36]), la réception de conseils sur l´AME lors des CPN (OR=2,92 IC 95% [1,70-5,01]), la réception de conseils sur l´AME lors des consultations post-natales (CPON) (OR=4,33 IC 95% [2,51-7,45]), la présence permanente de la mère à côté de son bébé (OR=3,97 IC 95% [1,99-7,91]) et l´existence de bonnes connaissances sur l´AME (OR=4,54 IC 95% [1,96-10,51]).

**Conclusion:**

ces facteurs modifiables peuvent permettre d´améliorer la pratique de l´AME dans la ville de Kaolack

## Abstract (en)

**Introduction:**

the World Health Organization (WHO) recommends immediate and exclusive breastfeeding in order to reduce malnutrition and neonatal mortality. The purpose of this study was to determine the prevalence of exclusive breastfeeding (EBF) and to identify its associated factors among mothers of children aged 6 to 12 months in Kaolack.

**Method:**

we conducted a cross-sectional descriptive and analytical study. The study was conducted from July 8^th^, 2020 to February 8^th^, 2021. Multivariate analysis was performed using the logistic regression model. The target population consisted of mothers of children aged 6 to 12 months residing in the city of Kaolack and met during vaccination sessions.

**Results:**

a total of 400 women were enrolled in the study. Among them, 51.8% practiced EBF according to the WHO recommendations. The average age of mothers was 27.42 years; 56.3% were housewives and 82.7% have done at least 4 antenatal care (ANC). Logistic regression showed an association between EBF and the following factors: age < 25 years (OR=2.03 CI 95% [1.23-3.36]), receiving advice on EBF during ANCs (OR=2.92 CI 95% [1.70-5.01]), receiving advice on EBF during postnatal care (PNC) (OR=4.33 CI 95% [2.51-7.45]), the permanent presence of mother with her baby (OR=3.97 CI 95% [1.99-7.91]), and a good knowledge of EBF (OR=4.54 CI 95% [1.96-10.51]).

**Conclusion:**

these modifiable factors can help improve the practice of EBF in the city of Kaolack.

## Introduction

En Afrique de l´Ouest, des progrès significatifs ont été notés dans la lutte contre la mortalité maternelle et infantile; mais la mortalité néonatale reste une source de préoccupation car ayant peu changé depuis 3 décennies [1,2]. Au Sénégal, la mortalité infantile est passée de 139‰ en 1997 à 37‰ en 2018, tandis que la mortalité maternelle est passée de 1992 à 2019 de 510 à 236 décès maternels pour 100000 naissances vivantes. La mortalité néonatale quant à elle, a peu évolué, passant de 37‰ en 1997 à 23‰ en 2018 [3-6]. L´allaitement précoce et exclusif représente l´une des interventions essentielles favorisant la survie néonatale [7]; on estime que 1,5 millions de ces décès dans le monde pourraient être évités par sa pratique [8]. L´allaitement maternel exclusif (AME) au sein est défini par l´OMS comme « la pratique de ne donner au nourrisson que du lait maternel au cours des 6 premiers mois; aucun autre liquide ou solide à l´exception de gouttes ou de sirops contenant des vitamines, des sels minéraux ou des médicaments ne peut être administré » [9]. L´OMS recommande sa poursuite jusqu´à 2 ans avec l´ajout d´aliments complémentaires appropriés. L´AME est parmi les différentes interventions préventives celle dont les effets sur la santé de la mère et de l´enfant sont les plus marqués [10]. L´allaitement maternel présente de nombreux avantages pour la santé de l´enfant et celle de sa mère. En effet, il favorise la croissance physique et affective de l´enfant, privilégie le lien mère-enfant, renforce l´immunité de l´enfant et réduit les coûts liés à l´achat de lait artificiel et au recours aux soins [7,11]. En 2012, l´OMS a fixé un objectif mondial en matière de nutrition consistant à augmenter le taux d´AME à 6 mois jusqu´à au moins 50% d´ici 2025. La pratique de l´AME reste encore non satisfaisante en dépit des politiques sanitaires mises en place. Au Sénégal, le taux d´AME chez les mères d´enfants de 6 moins est passé de 46% en 2018 à 41% en 2019 [5-6]. Beaucoup de facteurs sont associés à la pratique de l´AME en particulier les caractéristiques sociodémographiques, les facteurs liés à la santé de la mère et de l´enfant et l´offre de santé [12-16]. Ainsi, il nous parait nécessaire d´étudier les facteurs pouvant influencer et maintenir la pratique de l´AME au Sénégal, et plus précisément à Kaolack. Ce travail a pour objectif de déterminer la fréquence de la pratique de l´AME et d´identifier ses facteurs associés chez les mères d´enfants âgés de 6 à 12 mois dans la commune de Kaolack.

## Méthodes

**Cadre de l´étude**: le centre de santé (CS) de Kaolack est la structure de référence du district sanitaire de Kaolack qui est situé au centre du Sénégal à 192 km de Dakar. Le CS a pour zone de responsabilité la ville de Kaolack comptant 298859 habitants avec 69242 femmes en âge de procréer. La ville recèle en son sein une zone périurbaine où vivent des populations défavorisées. Il y existe plusieurs structures de santé dont l´hôpital de niveau 3, deux CS et plusieurs structures privées (cliniques, cabinets médicaux etc.). Ces structures mènent des activités surtout orientées vers la santé de la mère et de l´enfant, de médecine générale et de soins infirmiers. Le CS comporte : une maternité, un service de médecine, un cabinet dentaire, un service de vaccination, un service de consultation générale etc. Ces services offrent des prestations curatives et préventives (vaccination, consultations pré et post natale, dépistage VIH etc.) Le personnel est composé d´agents qualifiés (médecins, infirmiers, assistants-infirmiers, sages-femmes) mais aussi en grande partie d´agents non qualifiés (agents de santé communautaires, personnel de soutien, etc.).

**Contexte organisationnel:** il s’agit d’une étude transversale descriptive et analytique. La période d´étude s´étalait du 08 juillet 2020 au 08 février 2021 au niveau du centre de santé de Kaolack. La population cible de notre enquête était représentée par les mères d´enfants âgés de 6 à 12 mois résidant dans la ville de Kaolack rencontrées lors des séances de vaccination au niveau du centre de santé.

**Participants:** les critères d´inclusion étaient : être mère d´enfant âgé de 6 à 12 mois au moment de l´enquête, résider dans la ville et fréquenter les services de vaccination infantile du centre de santé durant la période de l´enquête. Les critères de non d´inclusion étaient: le refus, l´existence de trouble du langage. Un pas de sondage a été déterminé. Ainsi 20 femmes éligibles étaient recrutées et interviewées pendant les jours de vaccination du centre de santé qui se tenaient 5 fois par semaine.

**Variables-sources de données:** la collecte des données a été faite à l´aide d´un questionnaire préalablement prétesté. Les données étaient recueillies au cours d´un entretien avec les mères. Le carnet de santé de la mère et de l´enfant avait aussi servi de source d´informations. La variable dépendante était la pratique d´AME. Les variables indépendantes étaient: a) caractéristiques sociodémographiques: l´âge de la mère, le lieu de résidence, le niveau d´instruction de la mère, la profession de la mère, le statut matrimonial, le niveau d´instruction du conjoint, le détenteur du statut du chef de ménage, le revenu mensuel du ménage, la taille du ménage, le sexe de l´enfant, le rang de naissance de l´enfant, l´existence d´une allocation de bourse de sécurité familiale, et l´existence d´une couverture sanitaire; b) caractéristiques liées à l´alimentation du nouveau-né : le temps de présence de la mère avec le bébé, le délai entre la naissance et la prise de la première tétée, la prise d´aliments par le bébé avant la première tétée, l´âge du bébé à la première prise d´aliments autre que le lait maternel, l´existence chez la mère de bonnes connaissances sur l´AME, et le nombre de sources d´information sur l´AME reçues par la mère ; c) caractéristiques liées à la santé de la mère et de l´enfant: le nombre d´accouchements, le nombre de consultations prénatales (CPN) effectués, le nombre d´enfants vivants, le poids du bébé à la naissance, la réception de conseils lors des CPN, réception de conseils lors des consultations post-natal (CPoN), le lieu d´accouchement, le mode d´accouchement, le délai entre la naissance et la prise de vaccin contre l´hépatite B, le délai optimal de prise de la 3^e^ dose du vaccin Penta. Les connaissances des mères sur l´allaitement étaient évaluées à l´aide de 10 questions. Le score maximal attendu était de 10 points. Le niveau de connaissance était ainsi classé: mauvais (0-2 points), insuffisant (3-4 points), moyen (5-6 points), bon (7-10 points).

**Biais:** nos données viennent essentiellement des interviews des femmes. A cet égard nous pouvons avoir des biais de mémorisation. Pour réduire les biais de mémorisation nous avons choisi pour cible seulement les mères d´enfants âgés entre 6 et 12 mois. Au-delà de cet âge nous pouvons avoir des biais de mémorisation. Les biais de confusion éventuels peuvent être réduits par notre méthode d´analyse: la régression logistique.

**Taille de l´étude:** la taille de l´échantillon est calculée selon la formule de Schwartz: les hypothèses suivantes étaient posées: a) p correspond au taux de l´AME. Ce taux étant inconnu, il a été supposé que 41% des mères pratiquent l´AME; cette valeur représentant la moyenne nationale [6] b) ε = 1.96 pour un risque de première espèce Þ =0.05. i= précision=0.05. La taille de l´échantillon était égale à 372 et arrondie à 400.


$$n=\varepsilon^{2}.\frac{p\left(1-p\right)}{\iota^{2}}$$

**Variables quantitatives:** les variables quantitatives utilisées dans notre étude sont l´âge et le nombre d´accouchement. La variable âge a été discrétisé après consultations de nombreuses études et avis de spécialistes en gynéco-obstétrique.

**Méthodes statistiques:** l´analyse des données a été faite par le logiciel R version R-4.0.3. L´analyse univariée s´est faite avec une étude descriptive des données (moyenne, fréquence, écart type etc.). L´analyse bi-variée a été effectuée avec le test d´indépendance de khi^2^ ou le test exact de Fisher si les conditions d´application du test de Khi^2^ n´étaient pas remplies. Ces tests ont permis d´identifier les variables d´intérêt. L´intervalle de confiance était de 95%, et un lien significatif et indépendant était établi lorsque la p-value est inférieure à 0,05. L´analyse multivariée a été réalisée en utilisant le modèle de régression logistique binaire ajusté à toutes les variables. La sélection des variables pour le modèle complet s´est faite avec les variables significatives (p ≤0,05 au test de Kh^2^ ou au test exact de Fisher) et les variables dont le p ≤ 0,25 à ces tests. Les coefficients et la significativité du modèle ont été ensuite déterminés. La significativité globale du modèle a été faite par le test du rapport de vraisemblance. L´adéquation du modèle de régression logistique a été étudiée, c´est à dire on a évalué la qualité d´ajustement du modèle aux données (Pseudo-R^2^) et apprécié son pouvoir discriminant (courbe ROC, AUC, index C).

**Considération éthiques:** les autorités sanitaires ont autorisé l´étude. La participation à l´étude était libre et volontaire, avec un consentement éclairé des enquêtées. Aucun préjudice ou avantage ne découlait de la participation ou non à cette étude. Les données étaient collectées de façon anonyme et confidentielle. Les résultats de l´étude ont été transmis aux autorités sanitaires locales.

## Résultats

**Participants:** au total 400 femmes ont été enquêtées. Toutes les femmes ont répondu favorablement à l´enquête. Les carnets de santé de tous les enfants étaient disponibles. Nous avons noté 204 femmes qui ont pratiqué l´AME soit une fréquence de 51,8% dans la commune de Kaolack.

### Données descriptives

**Profil de la population étudiée: caractéristiques sociodémographiques:** l´âge moyen des mères était de 27,42 ans avec un écart type de 5,96 ans et des extrêmes allant de 15 et 47 ans. L´âge médian était de 27 ans. Le Tableau 1 résume les caractéristiques sociodémographiques des femmes enquêtées. Nous avons noté que 90,5% des mères résidaient dans la zone urbaine de Kaolack et que 22% des mères n´étaient pas instruites. La proportion de femmes au foyer était de 56,3% et 96,50% des mères d´enfants étaient mariées. Les ¾ des partenaires-mari étaient instruits et essentiellement de niveau secondaire 32%. Le chef de ménage était le plus souvent le mari (46,5%). Près de ¾ (73%) des ménages avaient des revenus inférieurs à 100000 frs CFA. La taille moyenne des ménages était de 9,98 personnes avec un écart type de 6,27. Plus des ¾ des ménages (78,50%) comptaient plus de 5 personnes avec des extrêmes de 1 et 40 et une médiane à 10. Le rang de naissance moyen était de 2 avec des extrêmes de 1 et 9^e^ rang et une médiane à 2. En ce qui concerne l´assistance sociale, on notait que 8% des femmes disposaient de bourse de sécurité sociale et 27,5% de couverture sanitaire.

**Tableau 1:** caractéristiques sociodémographiques des femmes enquêtées (n=400)

Variables	Effectifs (n)	Fréquences (%)
Age	24	6,7
	78	21,8
	100	28,0
Lieu de résidence	88	24,6
	49	13,7
Niveau d´instruction de la mère	16	4,5
	2	0,6
	357	100

**Caractéristiques liées à l´alimentation de l´enfant:** la totalité des enfants de notre série avaient été allaités au sein. Cependant, l´âge à la première prise d´aliments autre que le lait maternel était renseigné par 394 mères dont la moyenne était de 4,15 mois avec un écart type de 2,25 et des extrêmes de 1 et 8 mois. La médiane était de 6 mois. L´AME était effectué par 204 femmes (51,8%) et 190 femmes (47,5%) avaient donné à l´enfant des aliments autre que le lait maternel avant 6 mois. Les caractéristiques liées à l´alimentation de l´enfant sont décrites dans le Tableau 2. La majorité des mères (85%) étaient toujours avec leur nourrisson. Plus de ¾ des nouveau-nés (76,5%) sont allaités dans les 24 heures après leur naissance et 3,75% dans l´heure qui suit la naissance. Nous avons noté que 94,3% des mères avaient donné le colostrum à leur nourrisson. Le score moyen de la connaissance sur l´AME était de 8,21±1,43 et des extrêmes de 2 et 10 points. Le mode et la médiane étaient respectivement de 9 et 8 points. Nous avons noté que 88% des mères avaient une bonne connaissance de l´allaitement maternel et 10% une connaissance moyenne. L´étude a montré que 94,8% des mères avaient reçu des informations sur l´allaitement. La sage-femme (83,4%) était la principale source d´information sur l´allaitement suivie de la télévision (39,8%), de l´entourage (37,5%) et de la radio (23,7%). Cependant 36,5% des mères ont eu des informations sur l´allaitement à travers qu´une seule source.

**Tableau 2:** caractéristiques liées à l´alimentation de l´enfant

Variables	Effectifs (n)	Fréquences (%)
Temps de présence de la mère avec le bébé	133	37,25
	Moyenne: 16,73 ± 2,16 ans	
Délai entre la naissance et le prise de la première tétée	153	42,85
	Moyenne: 18,92 ± 3,44 ans	
	169	47,33
	11	6,5
Prise d´aliments par le bébé avant la première tétée	169	93,5
	166	46,49
Prise de colostrum par le bébé	73	44
	93	56
Age du bébé à la première prise d´aliments autre que le lait maternel	324	90,75
	316	97,5
Existence de bonnes connaissances sur l’AME	8	2,5
	298	83,47
Nombre de sources d´information sur sur l’AME	255	85,6
	23	7,7

**Caractéristiques liées à la santé de la mère et de l´enfant:** les principales caractéristiques liées à la santé de la mère et de l´enfant sont résumées dans le Tableau 3. Le nombre moyen d´accouchement était de 2,57 avec un écart type de 1,60 et des extrêmes de 1 et 9. La médiane était de 2. Le nombre moyen d´enfants des mères était de 2,44 avec un écart type de 1,45 et des extrêmes de 1 et 9 enfants. La médiane était de 2 enfants. Le poids moyen de naissance était de 3077 grammes avec un écart type de 493 et des extrêmes de 1500 et 5000 grammes. La quasi-totalité des femmes (99,3%) avaient bénéficié de CPN. Le nombre de CPN a été noté dans les carnets de santé chez 214 femmes avec une moyenne de 4,12 CPN, un écart type de 1,21 une médiane à 4 et des extrêmes de 1 et 9 CPN. L´étude a montré que 82,7% avaient effectué au moins 4 CPN. Plus de la moitié des femmes (60,7%) avaient reçu des conseils sur l´AME durant les CPN. L´étude a montré que 91,0% des mères avaient bénéficié de CPoN. Parmi ces dernières, 67,9% avaient reçu des conseils sur l´AME. Nous avons noté que 98% des femmes avaient accouché dans une structure sanitaire et 93,5% avaient accouché par voie basse. L´étude a révélé que 96% des enfants avaient été vacciné à la naissance contre l´hépatite B avec un délai moyen de 2,41 jours, une médiane à 0 jour, un écart type de 7,0 et des extrêmes allant de 0 et 62 jours. Le taux de Penta3 était de 97,8% avec un délai moyen de 3,95 mois, un écart type de 0,8 et des extrêmes de 1,6 et 9 mois.

**Tableau 3:** caractéristiques liées à la santé de la mère et de l´enfant

Variables	Modalités	Nombre (n)	Pourcentage (%)
Nombre d´accouchements	1	135	33,75
	2-3	165	41,25
	4-5	76	19
	≥ 6	24	6
Nombre d´enfants vivants	1	137	34,25
	2-3	181	45,25
	4-5	63	15,75
	≥ 6	19	4,75
Poids de naissance de léenfant	< 2500 gr	39	9,75
	≥ 2500 gr	361	90,25
Réception ou non de conseils sur l´AME lors des CPN	Non	156	39,3
	Oui	241	60,7
Réception ou non de conseils sur l´AME lors des CPoN	Non	117	32,1
	Oui	247	67,9
Lieu d´accouchement	Domicile	8	2
	Structure de santé	392	98
Modes d´accouchement	Césarienne	26	6,5
	Voie basse	374	93,5
Délai entre la naissance et la prise de la vaccination contre l´hépatite B	≤ 24 h	321	80,25
	> 24 h	79	19,75
délai optimal de prise de la 3^è^ dose du vaccin Penta	oui	391	97,75

**Résultats de l´analyse: analyse bivariée:** une analyse bi-variée a été réalisée afin d´identifier les variables d´intérêt (Tableau 4). Nous avons mis en évidence une association statistiquement significative (p < 0,05) entre l´AME et les facteurs suivants: la profession de la mère, la réception de conseils sur l´AME lors des CPN, la réception de conseils sur l´AME lors des CPoN, le nombre de sources d´information sur l´AME, la présence de la mère avec le bébé, la prise ou non d´aliments par le bébé avant la première tétée, la prise ou non du colostrum par le bébé et l´existence ou non de bonnes connaissances de la mère sur l´AME et le nombre de sources d´informations sur l´allaitement reçues par la mère ≥ 2. Les variables significatives aux tests (p<0,05) et les autres variables qui ont des p-values < 0,2 aux tests (âge des femmes, taille des ménages) ont été intégrées au modèle complet. L´analyse des variables n´a pas permis de retrouver des interactions et des facteurs de confusion.

**Tableau 4:** résultats de l´analyse bivariée

Variables	Effectif et % de femmes ayant fait l’AME	P value
Age des femmes < 25 ans	80 (55,56%)	0,172
Lieu de résidence Urbain	186 (51,4%)	0,638
Mères instruites	156(50%)	0,451
Mères sans profession	141 (56,4%)	0,005
Conjoints instruits	154(51,1%)	0,909
Mari-femme chef de ménage	13(41,9%)	0,293
Revenu mensuel du ménage < 100000 francs CFA	147 (50,3%)	0,665
Nombre d’accouchements = 1	64 (47,4%)	0,305
Nombre d’enfants vivants = 1	65 (47,4%)	0,271
Taille du ménage ≤ 5 personnes	51(59,3%)	0,08
Enfant de sexe féminin	111(53,4%)	0,325
Poids de naissance de l´enfant < 2500 gr	16(41%)	0,189
Rang de naissance de l’enfant 1er rang	64 (46,7%)	0,216
Absence d’allocation d’une bourse de sécurité familiale	185 (50,4%)	0,430
Absence de couverture sanitaire	149 (51,4%)	0,805
Réception de conseils sur l´AME lors des CPN	161(66,8%)	0,000
Accouchement dans des structures de santé	200(51,1%)	0,691
Accouchement par voie basse	193(51,6%)	0,74
Réception de conseils sur l’AME lors des CPoN	167(67,6%)	0,000
Délai vaccination Hépatite B ≤ 24 h	168(52,3%)	0,281
Délai optimale de prise du vaccin Penta 3	75(49,3%)	0,604
Nombre de sources d´informations sur l´AME ≥ 2	145(57%)	0,001
Mère toujours avec le bébé	187 (55%)	0.0
Prise immédiate de la première tétée ≤ 1 heure	7 (46,7%)	0,73
Non prise d’aliments avant la première tétée	115 (59,3%)	0,001
Prise de colostrum	197(52,2%)	0,042
Bonnes connaissances sur l´AME	195(55,4%)	0,0
Sources d´information ≥2	145(57%)	0,001

### Principaux résultats

**Analyse multivariée:** l´analyse multivariée a permis d´identifier les facteurs de risque associés à la pratique de l´AME. Le modèle final est constitué des variables significatives au test de Wald. Il s´agit de: l´âge < 25 ans (OR=2,03 IC 95% [1,23-3,36]), la réception de conseils sur l´AME lors des CPN (OR=2,92 IC 95% [1,70-5,01]), la réception de conseils sur l´AME lors des CPoN (OR=4,33 IC 95% [2,51-7,45]), la présence permanente de la mère à côté de son bébé (OR=3,97 IC 95% [1,99-7, 91]) et l´existence de bonnes connaissances sur l´AME (OR=4,54 IC 95% [1,96-10,51]).

**Autres analyses:** la significativité globale du modèle a été déterminée par le test du maximum de vraisemblance qui a mis en évidence une différence significative entre le modèle retenu et le modèle constitué uniquement par le facteur étudié (χ^2^ = 49.176 et p=0,000). Les variables explicatives ont simultanément une influence sur la probabilité d´apparition de l´évènement. La qualité d´ajustement global du modèle a été évalué par le Pseudo-R^2^ de Mc Fadden qui est égal à 0,46. Il est jugé significatif pour cette série (p-value > 0,2). L´aire sous la courbe ROC (AUC) est une mesure du pouvoir discriminant du modèle. Avec un AUC égal à 0.84, notre modèle a une discrimination excellente. La capacité prédictive du modèle a aussi été mesurée par le l´index C (probabilité de concordance) qui est égal 0,84; notre modèle est par conséquent excellent selon la classification. Le test des Résidus de Pearson (p=1) qui sert à évaluer la pertinence du modèle, met en évidence des résultats significatifs (p > 0,05); le modèle est bien adapté aux données.

## Discussion

### Résultats clés

**Prévalence de l´allaitement maternel exclusif:** au Sénégal, la pratique de l'allaitement est courante. Selon l'EDS 2016, 99 % des femmes sénégalaises donnent le sein. Ces résultats sont corroborés par notre étude qui montre que la totalité des mères pratiquent l´allaitement. Cependant, cette pratique s'accompagne de l'administration d'eau et d'autres liquides bien avant le sixième mois. Cette étude a montré que 51.8% des mères pratiquaient l´AME. Ce taux est supérieur au taux national qui est de 41% en 2019 [6]. Ce taux est également supérieur à l´objectif de l´OMS qui en 2012 voulait porter le taux d´AME au cours des six premiers mois jusqu´à au moins 50 % d´ici 2025 [17]. Ce taux supérieur à l´objectif national et mondial peut s´expliquer par le fait que Kaolack soit une ville où l´offre de santé est importante et les moyens de communication sont plus accessibles. Les structures de santé offrent des prestations préventives (CPN, CPoN, vaccination) qui sont des occasions pour le personnel de donner des informations relatives à l´AME. En outre, il existe en ville plus de disponibilité d´informations sanitaires en raison de nombre de structures de santé important et de la diversité des canaux d´information (radio, télévision, réseau communautaire etc.) Ce taux trouvé à Kaolack est élevé par rapport aux résultats de l´EDS 2019 qui montre un taux d´AME de 41% au Sénégal [18]. Une étude similaire réalisée dans la ville de Thiès montre un taux d´AME de 41,5% [19]. En raison du monde d´échantillonnage de l´EDS, le taux d´AME trouvé indique une prévalence nationale et ne reflète pas donc les disparités régionales. En Ethiopie une étude a montré un taux de 44,2% ; cela était inférieur à la valeur de leurs EDS en 2005, qui était de 49% [20]. Au Mali, une étude a révélé un taux de 30,66% [21] supérieur au taux de 20,4% retrouvé dans l´Enquête par Grappes à Indicateurs Multiples 2009 - 2010 du Mali en 2010 [22]. En Afrique, la prévalence de l´AME de plusieurs pays est en dessous des recommandations de l´OMS. Une étude faite au Burkina Faso abonde dans ce sens avec un taux de 40,0% en 2017 [15,20,23].

**Limites de l´étude:** cette étude est la première faite dans ce district sanitaire dans le domaine de l´allaitement maternel. Par conséquent elle pourrait être une référence pour les études à venir. Nous avons eu recours à une régression logistique lors de l´analyse nous permettant ainsi de neutraliser les facteurs de confusion. Néanmoins, elle présente trois limites. La première est liée à un biais de mémorisation qui pourrait être induit par le fait que la date d´arrêt de l´AME (introduction d´aliments autre que le lait maternel avant l´âge de 6 mois dans l´alimentation du bébé) était établi sur la base de la déclaration de la mère. Cependant, l´intervalle entre la période de l´enquête et l´arrêt éventuel de l´AME est relativement court; cela a permis de réduire le risque de survenue de ce biais. La deuxième limite est liée à un biais de sélection. En effet, la population d´étude était constituée des mères qui faisaient vacciner leurs nourrissons au CS de Kaolack. Les mères qui ne fréquentent pas le CS ou qui ne font pas vacciner leurs nourrissons étaient exclues de l´étude. La troisième limite est liée au fait que les résultats sont principalement valables que dans le contexte la ville de Kaolack ou dans des localités similaires.

**Interprétation: facteurs associés la pratique de l´AME**: les facteurs retenus à la suite de la régression logistique sont les suivants: l´âge < 25 ans, la réception de conseils sur l´AME lors des CPN, la réception de conseils sur l´AME lors des CPoN, la présence permanente de la mère à côté de son bébé et l´existence de bonnes connaissances sur l´AME.

**Age de la mère:** notre étude a trouvé une association significative entre la pratique de l´AME et l´âge de la mère. Les mères âgées de moins 25 ans ont 2,03 fois plus de chance de pratiquer l´AME que celles qui ont plus de 25 ans (OR: 2,03 IC:[1,23-3,36] p : 0,006). Ces résultats s´expliquent par le fait que l´ancienne génération est plus axée sur les traditions. La nouvelle génération fait plus confiance à internet. Elle a tendance à faire des recherches pour être plus en phase avec les nouvelles recommandations concernant leur santé et celle de leurs enfants. Durant la collecte, de jeunes mères instruites ont révélé avoir lu le carnet de santé mère-enfant durant leur grossesse et y ont trouvé l´importance de la pratique de l´AME. Une étude faite par Sangho H *et al*. en 2009 abonde dans ce sens en indiquant que l´âge plus jeune (moins de 30 ans) des mères était favorable à l´AME [21]. Par contre, d´autres études ont mis en évidence une situation contraire. En Espagne, Gonzalez *et al*. ont montré que l´âge de plus de 35 ans était associé au démarrage et au maintien de l´AME [24]. Dans ce dernier cas, il faut reconnaitre que les contextes socio-économiques sont totalement différents avec celles des pays en voies de développement.

**Réception de conseils sur l´AME lors des CPN:** les conseils reçus lors des CPN ont influencé positivement la pratique de l´AME. Les mères qui avaient reçu l´information lors des CPN ont 2,92 fois plus de chance de pratiquer l´AME que celles qui n´en ont pas reçue (OR :2,92 IC: [1,70-5,01] p: 0,001). Cette étude montre qu´un nombre optimal de CPN dépourvues d´éducation prénatale n´est pas positivement associée à la pratique de l´AME. Ainsi, un nombre élevé de CPN sans délivrance de conseils sur l´allaitement ne serait lié à la pratique de l´AME. Selon T. Hanane, les mères qui ont reçu des conseils durant les CPN ont deux fois plus de chance de pratiquer l´AME [25]. De même, Duenas-Espin Ia indiqué que l´éducation prénatale réduit de 30% le risque de cessation de l´AME avant l´âge de six mois [26]. Mouhamed AI *et al*. au Somaliland et, Tsegaw SA *et al*. en Ethiopie ont constaté que les femmes ayant reçu de l´information sur l´allaitement lors des CPN sont plus sujettes à pratiquer l´AME [27,28].

**Réception de conseils sur l´AME lors des CPoN:** cette étude a mis en évidence une relation entre l´AME et les conseils reçus lors des CPoN. Les mères qui avaient reçu des conseils sur l´allaitement lors des CPoN avaient 4,33 fois plus de chance de pratiquer l´AME (OR: 4,33 IC: [2, 51-7,45] p: 0,001). L´OMS inclut dans le paquet d´informations à donner à la CPoN des conseils relatifs à l´AME [17].

**Temps passé avec le nourrisson:** les mères qui étaient tout le temps avec leurs nourrissons ont 3,97 fois plus de chance de réussir l´AME (OR:3,97 IC:[1,99-7,91] p: 0,001). Pour passer du temps avec son enfant, il faut être femme au foyer ou avoir un travail peu contraignant. Des professions comme fonctionnaire et élève/étudiant avec des horaires stricts ne permettent pas de rester beaucoup de temps avec son enfant et donc de pratiquer l´AME. En Côte d´Ivoire, Coulibaly a également trouvé que les mères fonctionnaires et élevé/étudiante ne pratiquaient pas l´AME [13]. Par contre, selon Robert E en Wallonie (Belgique), les femmes travailleuses allaitent le plus souvent comparées à leurs homologues ménagères [16]. Cette tendance est confirmée par d´autres études: Bonet M en France [29], et Sittlington J *et al*. en Irlande du Nord [30].

**Existence de bonnes connaissances sur l´AME:** cette étude a montré que les mères qui ont des bonnes connaissances sur l´allaitement avaient 4,54 fois plus de chance de pratiquer l´AME (OR: 4,54 IC: [1,96-10,51] p: 0,001). Ce résultat apporte la preuve de l´importance de l´éducation des mères à l´AME. Plusieurs études conduites respectivement au Mali, en Espagne et en Chine ont trouvé une relation favorable entre la pratique de l´AME et les connaissances sur l´allaitement [21,31-34]. La connaissance des modalités de l´AME joue un rôle potentiel dans sa réussite [34].

**Généralisibilité:** la troisième limite de notre étude est que ses résultats ne peuvent pas être généralisés à l´ensemble du pays en raison d´une part des limites précédemment citées et d´autre part par le caractère urbain de notre zone d´étude. En effet il existe une grande différence entre les zones rurales et urbaine en matière d´offre de santé, instruction des populations, pouvoir économique etc. Néanmoins ces résultats peuvent être généralisés aux zones qui ont les mêmes caractéristiques que la ville de Kaolack.

## Conclusion

La pratique de l´AME reste satisfaisante dans la ville de Kaolack avec un taux de 51,8; taux supérieur aux objectifs de l´OMS (50% en 2025). Notre étude a montré que plusieurs facteurs étaient significativement associés à cette pratique: l´âge < 25 ans, la réception de conseils sur l´AME lors des CPN, la réception de conseils sur l´AME lors des CPoN, la présence permanente de la mère à côté de son bébé et l´existence de bonnes connaissances sur l´AME. Les autorités sanitaires devraient prendre en compte ces facteurs pour améliorer d´avantage la pratique de l´AME notamment en renforçant la communication en direction des femmes en âge de procréer et en donnant des conseils sur l´allaitement lors des CPN et des CPoN.

### Etat des connaissances sur le sujet

- *La connaissance de la technique de l´AME et de ses bienfaits pour la santé de la mère et de l´enfant qui un effet positif sur la l´AME;*
- *La source d´information les plus déterminante reste le personnel de santé qui profite des contacts avec la femme enceinte ou récemment accouchée pour prodiguer des conseils sur l´AME*.

###  Contribution de notre étude à la connaissance

- *La ville de Kaolack a atteint avant l´échéance le taux d AME fixé par l´OMS pour 2025 (taux AME 50% en 2025);*
- *L´âge des femmes < 25 ans est un facteur associé à une meilleure pratique de l´AME. Ceci est très rarement retrouvé dans les études réalisées en Afrique;*
- *Le niveau d´instruction ne joue pas un rôle dans la pratique de l´AME*.
